# Guillain-Barre Syndrome Caused by Mycoplasma pneumoniae Infection in an Elderly Patient Initially Misdiagnosed As Frailty

**DOI:** 10.7759/cureus.22386

**Published:** 2022-02-19

**Authors:** Morika Suzuki, Genya Watanabe, Takashi Watari

**Affiliations:** 1 Department of General Internal Medicine, National Hospital Organization Sendai Medical Center, Miyagi, JPN; 2 Department of General Neurology, National Hospital Organization Sendai Medical Center, Miyagi, JPN; 3 Division of Hospital Medicine, University of Michigan Health System, Ann Arbor, USA; 4 General Medicine Center, Shimane University Hospital, Izumo, JPN

**Keywords:** differential diagnoses of frailty, mycoplasma pneumoniae infection, diagnostic error, frailty, guillain-barré syndrome

## Abstract

A 73-year-old man with a history of hypertension was referred to our department because of progressive frailty while being treated for acute heart failure. Physical examination revealed loss of tendon reflexes in the lower extremities and muscle weakness in all extremities. After close examination, he was diagnosed with Guillain-Barre syndrome (GBS), which was preceded by a Mycoplasma pneumoniae infection. He was given high-dose intravenous immunoglobulin (IVIg) therapy, and his muscle weakness improved.

This case suggests that symptoms of neurological disorders in the elderly may be viewed as frailty. Being elderly is a poor prognostic factor for GBS; therefore, early diagnosis and consultation with a neurologist are necessary. Physical examination is essential to differentiate frailty from neurological disorders, and deep tendon reflexes are instrumental in making a diagnosis of GBS.

## Introduction

In recent years, the population of elderly people has increased worldwide, and Japan has the highest proportion of elderly people in the world. Frailty is defined as a state in which elderly individuals can only maintain and improve their life functions with appropriate intervention and support [1]. Hospitalization for inpatient care is more likely to lead to the progression of frailty, as the physical and mental vulnerabilities of the elderly become more pronounced in such conditions due to environmental changes and illnesses. However, symptoms of neurological disorders may manifest as muscle weakness and gait disturbance and must be differentiated from frailty.

Guillain-Barre syndrome (GBS) is a rapidly progressive polyneuropathy that results in progressive muscle weakness and loss of tendon reflexes. It is often associated with prior infections, such as gastroenteritis and upper respiratory tract infections, along with diarrhea. Campylobacter jejuni is the most common source of infection in such cases, but Cytomegalovirus (CMV), Epstein-Barr virus (EBV), and Mycoplasma (M. pneumoniae) are also causative agents. GBS is generally considered to have a good prognosis, as it remits in about a year, but severe movement disorders may persist. Thus, the inability to walk unsupported six months after the onset of the disease is considered a poor prognostic factor, and advanced age and rapid progression of muscle weakness can further contribute to the poor prognosis [2].

In this study, we present a case of GBS after Mycoplasma pneumoniae infection (MPI) in an elderly person misdiagnosed with frailty. We hypothesize that failure to physically evaluate neurological symptoms in elderly patients may contribute to delayed diagnosis. Particularly, the presence of decreased tendon reflexes is important in differentiating GBS from frailty.

## Case presentation

A 73-year-old man with a history of hypertension was referred to the general internal medicine (GIM) department of our hospital due to the progression of frailty and weakness after the improvement of acute heart failure.

The patient had a fever and dry cough 21 days prior to admission to our department (PTA), and this was treated with antipyretics and antitussives. Subsequently, after 14 days PTA, he was referred to his previous doctor because of tachypnea and pedal edema. Blood tests at this time showed a mildly elevated inflammatory response and mildly elevated hepatobiliary enzymes. Additionally, computed tomography (CT) imaging showed no features of pneumonia. However, cardiac enlargement, bilateral pleural effusions, and pericardial effusion were noted. The patient was diagnosed with acute heart failure due to acute bronchitis and pericarditis and was admitted to our hospital under the supervision of the department of cardiology for treatment 12 days PTA. After treatment for acute heart failure using diuretics, his heart failure symptoms and cough and fever improved. However, his gait gradually became slower, and his grip strength (weakness, slowness, and exhaustion) decreased after hospitalization. As a result, he was diagnosed with frailty seven days PTA. He started rehabilitation seven days PTA, but from two days PTA 2, the patient experienced difficulty in walking independently and his condition deteriorated. At that time, his frailty was attributed to disuse due to exhaustion caused by infection and hospitalization for acute heart failure, and no neurological examination was performed.

His vitals at the time of his visit to the GIM department were as follows: temperature, 36.8°C; blood pressure, 130/68 mm Hg; pulse, 76 beats/min; respiratory rate, 12 breaths/min; and oxygenation, 98% in room air.

The jugular venous pressure was not elevated, his heartbeat was clear, and there were no heart murmurs on physical examination. There were no rales in the lung fields and no pedal edema. The neurological examination showed no cranial nerve abnormalities. On motor examination, the bilateral, distal, and upper and lower extremity manual muscle test result was reduced to 4, and grip strength was reduced to 24 on the right and 25 on the left. A sensory examination showed reduced superficial sensation of the distal upper and lower extremities, and a bilateral lower extremity vibration sensation was absent. Reflex testing showed loss of patellar tendon reflexes and Achilles tendon reflexes bilaterally.

Blood investigations showed features of inflammation such as elevated white blood cell (WBC) count (13400 cells/μL) and C-reactive protein (CRP) levels (6.7 mg/dL). Mild elevation in liver enzymes was also noted (aspartate transaminase/alanine transaminase: 59/75 mU/mL). Cerebrospinal fluid (CSF) examination showed no alterations in the initial pressure (130 mmHg) and cell count and CSF protein levels (27 mg/dL), with no protein-cell dissociation. The CSF glucose level was 40 mg/dL (serum blood glucose of 99 g/dL 3 hours before CSF sample collection (Table 1).

**Table 1:** Laboratory data

CBC/Biochemistry				
White blood cell	13.4	10³/μL		
Red blood cell	365	10³/μL		
Hemoglobin	11	mg/dL		
Platelets	539	10³/μL		
Urea	18	mg/dL		
Creatinine	0.8	mg/dL		
Aspartate transaminase (AST)	59	mU/mL		
Alanine aminotransferase (ALT)	75	mU/mL		
C-reactive protein	6.7	mg/L		
Glucose	99	mg/dL		
Cerebrospinal fluid results				
Appearance	Clear			
Initial pressure	130	mmHg		
Cells	<1	cells		
Protein	27	mg/L		
Glucose	40	mg/dL		
Serologise:blood				
Mycoplasma pneumonia				
IgM antibodies	Positive			
Particle agglutination level	2560	times higher than normal.		
Antiganglioside anibobies				
GM1, GD1b	Positive			
GM2, GM3, GD1a, GD3, GQ1b	Negative			

**Figure 1:**
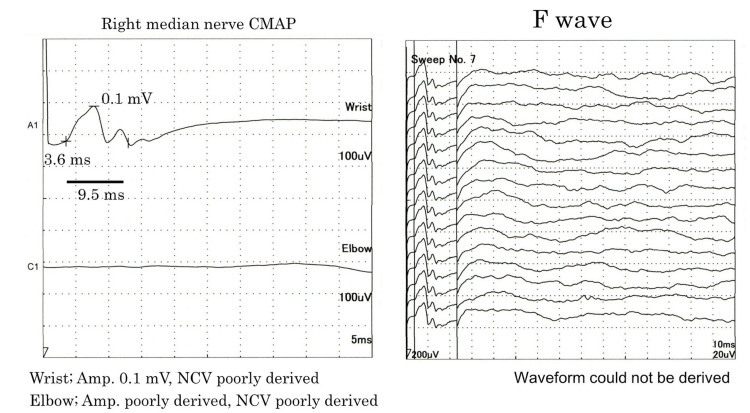
A nerve conduction velocity test In the complex muscle action potentials (CMAPs) of the right median nerve, the proximal waveform and F waves could not be derived and the amplitude of the distal waveform was significantly decreased.

Nerve conduction studies (NCS) failed to derive F waves in both the upper and lower extremities. The complex muscle action potential amplitude was significantly decreased in the median nerve and was not detected in the ulnar, tibial, and peroneal nerves (Figure 1).

The sensory nerve action potential (SNAP) amplitude of the median, ulnar, and sural nerves were not derived.

The patient was positive for M. pneumoniae IgM antibodies, and the mycoplasma particle agglutination level was 2560 times higher than normal. Furthermore, anti-ganglioside antibodies were positive for anti-GM1 IgG and anti-GD1b IgG, suggesting an acute motor and sensory axonal neuropathy (AMSAN) form of GBS.

Based on these results, a diagnosis of GBS was made with Mycoplasma as the predominant infection. His respiratory status was stable, and no dysphagia was observed. After intravenous immunoglobulin (IVIg) treatment, his muscle strength recovered, and he was able to write and walk. The patient was transferred to the hospital for rehabilitation because there were improvements in some waveforms.

Investigations

We suspected GBS due to decreased tendon reflexes in both legs. The spinal fluid analysis and NCS results were consistent with GBS. The initial infection was an upper respiratory tract infection, and elevated Mycoplasma antibodies were detected. The cause of the acute bronchitis was MPI, and epicarditis was thought to be an extrapulmonary complication of MPI. As an adjunct to GBS, testing for anti-ganglioside antibodies was performed, and the results were positive for anti-GM1 and anti-GD1b IgG, suggesting the AMSAN form of GBS.

We show here the differential diagnosis of frailty as a mimicker of GBS that should be considered in general clinical practice (Table 2).

**Table 2:** Differential diagnoses of frailty as a mimicker of GBS GBS: Guillain-Barre syndrome

Differential diagnoses of frailty as a mimicker of GBS								
Peripheral neuropathy: due to organic solvent poisoning, porphyrin metabolic disorders, diphtheria, lead poisoning, or vascular disorders								
Nutritional disorders								
Chronic inflammatory demyelinating polyneuropathy (first-ever, acute onset)								
Neurodegenerative disease: amyotrophic lateral sclerosis								
Neuromuscular junction disorders: myasthenia gravis, botulism, shellfish poisoning								
Muscle diseases: myositis, hypokalemia, hypophosphatemia, rhabdomyolysis, periodic tetraplegia								
Brain disorders: stroke, meningitis, demyelinating disease, brain tumor								
Psychiatric disorder: dissociative disorder								
Infectious diseases: polio, tick paralysis, West Nile virus								
Electrolyte abnormalities: metabolic disorders, hypokalemia, hypophosphatemia								
Malignant tumor: paraneoplastic neurological syndrome								

Treatment

Twenty days after the patient first visited our department, IVIg therapy was administered (400 mg/day/kg; for five days).

Outcome and follow-up

The patient's muscle strength gradually recovered after receiving IVIg. After 15 days of IVIg therapy (35 days after the patient first visited our department), the NCS result also showed the appearance of waveforms in the median nerve. One month after IVIg therapy (50 days after the patient first visited our department), the patient could walk (20 m with light assistance) and write. Two months after the first visit, the patient’s symptoms had improved to the point where the patient could walk with a cane, and he was transferred to the hospital for rehabilitation.

## Discussion

This was a case of GBS after MPI in an elderly patient that was misdiagnosed as frailty. The delay in diagnosis was due to failure in differentiating neurological symptoms on physical examination.

Frailty is defined as the emergence of physical and mental weaknesses with age, but with appropriate interventions and support, it is possible to maintain and improve the quality of life. Various methods have been proposed to assess frailty. However, Fried et al. proposed shrinking, weakness, exhaustion, slowness, and low activity as diagnostic criteria that are easily adaptable to clinical practice [1]. Symptoms are more likely to progress in an infectious or inpatient setting, and early hospital discharge and rehabilitation are effective [3-4].

While GBS is a rapidly progressive polyneuropathy, a set of diagnostic criteria have been developed to enable its diagnosis by non-specialists. If two criteria are met, namely, progressive muscle weakness in more than two limbs and loss of tendon reflexes, the patient should be evaluated further for GBS [5-6].

In general, GBS is a disease with a good prognosis because patients usually go into remission within a year, although severe motor deficits may persist. The prognosis is poor if the patient is unable to walk unaided six months after the onset of the disease. Early diagnosis is necessary because advanced age and rapid progression of muscle weakness are poor prognostic factors [2]. GBS is often associated with prior infections and MPI [7-8]. A test for GBS is known to show protein-cell dissociation of spinal fluid; however, spinal fluid proteins may not increase in the early stages of the disease, and retesting a few weeks later may show elevated CSF protein. Moreover, an adjunct diagnosis with anti-ganglioside antibodies may be helpful [9].

This case was diagnosed as frailty because of weakness and exhaustion during hospitalization for heart failure. We diagnosed this patient with GBS after MPI and administered IVIg, which improved muscle strength. While undergoing treatment for heart failure, GBS symptoms, such as loss of grip strength and difficulty in walking gradually progressed, but the initial diagnosis of frailty delayed GBS diagnosis. This case suggests that neurological symptoms in the elderly may be mistaken as frailty. Advanced age is a poor prognostic factor for GBS; therefore, early consultation with a neurologist is necessary. Physical examination is vital to differentiate frailty from neurological disease, and tendon reflexes are instrumental in making a diagnosis of GBS.

## Conclusions

Neurological symptoms in the elderly may be viewed as frailty, and GBS in the elderly is a poor prognostic factor, hence, early diagnosis is essential. We believe that failure to physically evaluate the neurological symptoms in elderly patients may contribute to delayed diagnosis. The presence or absence of decreased tendon reflexes is a high-yield finding differentiating GBS from frailty.
